# Novel biomarker genes for the prediction of post-hepatectomy survival of patients with NAFLD-related hepatocellular carcinoma

**DOI:** 10.1186/s12935-023-03106-2

**Published:** 2023-11-10

**Authors:** Yuting Song, Ying Wang, Xin Geng, Xianming Wang, Huisi He, Youwen Qian, Yaping Dong, Zhecai Fan, Shuzhen Chen, Wen Wen, Hongyang Wang

**Affiliations:** 1 Model Animal Research Center, Nanjing University, Nanjing, 210008 China; 2 National Center for Liver Cancer, Naval Medical University, Shanghai, 201805 China; 3 International Cooperation Laboratory on Signal Transduction, Third Affiliated Hospital of Naval Medical University, Shanghai, 200438 China; 4 Department of Laboratory Medicine, Shanghai Eastern Hepatobiliary Surgery Hospital, Shanghai, 200438 China

**Keywords:** Nonalcoholic fatty liver disease related hepatocellular carcinoma, RNA-Seq, Text-mining, FABP4, VWF

## Abstract

**Background:**

The incidence and prevalence of nonalcoholic fatty liver disease related hepatocellular carcinoma (NAFLD-HCC) are rapidly increasing worldwide. This study aimed to identify biomarker genes for prognostic prediction model of NAFLD-HCC hepatectomy by integrating text-mining, clinical follow-up information, transcriptomic data and experimental validation.

**Methods:**

The tumor and adjacent normal liver samples collected from 13 NAFLD-HCC and 12 HBV-HCC patients were sequenced using RNA-Seq. A novel text-mining strategy, explainable gene ontology fingerprint approach, was utilized to screen NAFLD-HCC featured gene sets and cell types, and the results were validated through a series of lab experiments. A risk score calculated by the multivariate Cox regression model using discovered key genes was established and evaluated based on 47 patients’ follow-up information.

**Results:**

Differentially expressed genes associated with NAFLD-HCC specific tumor microenvironment were screened, of which FABP4 and VWF were featured by previous reports. A risk prediction model consisting of FABP4, VWF, gender and TNM stage were then established based on 47 samples. The model showed that overall survival in the high-risk score group was lower compared with that in the low-risk score group (p = 0.0095).

**Conclusions:**

This study provided the landscape of NAFLD-HCC transcriptome, and elucidated that our model could predict hepatectomy prognosis with high accuracy.

**Supplementary Information:**

The online version contains supplementary material available at 10.1186/s12935-023-03106-2.

## Introduction

Liver cancer is predicted to be the sixth most commonly diagnosed cancer and the third leading cause of cancer death worldwide. Liver cancers consist of 85–90% hepatocellular carcinoma (HCC) and 10–15% cholangiocarcinoma [1]. HCC has several known risk factors, including chronic liver disease and liver cirrhosis caused by hepatitis B or C virus (HBV or HCV) infection, alcohol abuse and metabolic syndrome [2, 3]. Universal HBV vaccination and wide implementation of direct-acting antiviral agents against HCV are likely to change the etiologic landscape of hepatocellular carcinoma. NAFLD is defined as liver fat accumulation in more than 5% of hepatocytes without HBV/HCV infection or excessive alcohol consumption. Recently, nonalcoholic fatty liver disease (NAFLD) has been recognized as the most common chronic liver disease worldwide [4]. Owing to the increasing prevalence, NAFLD is predicted to become a leading cause of HCC soon [5]. Currently, the main treatments for HCC patients in early stages are curative resection, liver transplantation, radiofrequency ablation, trans-arterial chemoembolization, radioembolization and systemic targeted agent like sorafenib [6, 7]. Because the number of NAFLD-HCC patients is smaller compared with that of HBV-HCC currently, and the NAFLD-HCC patients are usually also infected with HBV, the recruitment of NAFLD-HCC patients is difficult. In addition to the lack of follow-up information, the biomarker for prognostic prediction model for those NAFLD-HCC treatments was rarely reported.

The progression from nonalcoholic steatohepatitis (NASH) to HCC is strongly influenced by the composition and abundance of different cell types in the tumor microenvironment [8]. For example, hepatic stellate cells are critical for driving liver fibrosis [9], and the inhibition of lipid and cholesterol synthesis in hepatic stellate cells may be an effective approach for mediating the anti-fibrotic effects [10]. Diet-induced NASH was characterized by the induction of tumor-associated macrophage-like macrophages and exhaustion of cytotoxic CD8 + T cells in the liver [11]. In addition, the clinical significance of the stromal and immune cells in the liver cancer microenvironment is supported by increasing evidence [12–14]. For example, angiogenesis, immune suppression, chemotherapeutic resistance, and tumor cell migration are related to stromal cells and immune cells that infiltrate tumors [12, 15]. Nevertheless, the microenvironmental biomarker in combination with NAFLD-HCC prognosis has been rarely reported yet.

Recent progress in omics technologies such as transcriptomics offers an unprecedented opportunity to understand the disease mechanisms, including the effect of tumor microenvironment. Computational algorithms could be used to infer microenvironment cell composition from bulk tumor transcriptome profiles [16]. These estimated tumor microenvironments inferred from transcriptome in the tumors provided insight into tumor–microenvironment interactions, and those genes associated with tumor microenvironment may serve as novel biomarkers for cancer development [16].

In this study, we compared the transcriptomes from tumor and adjacent normal samples of HCC patients with only either NAFLD or HBV who underwent tumor resection and exhibited distinct overall survival in our follow-up. Meanwhile, tremendous data available in the biomedical literature and a new method explainable gene ontology fingerprint (XGOF) developed in our previous study for text-mining [17] help to screen potential key genes from the different expressed genes (DEGs) in the transcriptome. By integrating transcriptome and text-mining results, we sought to comprehensively decipher molecular and microenvironmental differences between the two HCC types. At last, we constructed a multi-feature joint model to predict the prognosis of NAFLD-HCC.

## Method

### Patients and Samples

NAFLD-HCC patients who underwent no treatment before recruited and a subsequent liver resection at Shanghai Eastern Hepatobiliary Surgery Hospital were included in this study. We strictly screened clinical hepatocellular carcinoma samples to exclude viral hepatitis and schistosomiasis. Since HCC was mainly caused by HBV infection in China, HBV-HCC was set as a control group in this study. Totally, 25 pairs of matched pairs of primary HCC samples and adjacent normal liver tissue were collected including 13 NAFLD-related HCC and 12 HBV-related HCC (Table S1). The patients were followed up for up to 9 years. Overall survival (OS) was defined as the interval between the date of surgery and the date of patient death or last follow-up. Informed consent was obtained from all patients included in the study prior to surgery. The present study was approved by the Institutional Ethics Review Board of Shanghai Eastern Hepatobiliary Surgery Hospital. All procedures followed were in accordance with the ethical standards of the responsible committee on human experimentation and with the Helsinki Declaration of 1975, as revised in 2000.

### Transcriptomic sequencing and data analysis

A total of 25 tumor and adjacent normal liver samples collected from 13 NAFLD-HCC and 12 HBV-HCC patients were sequenced using RNA-Seq. DESeq2 was used to analyze DEGs based on the threshold criteria of log_2_ fold change > 1 or < -1 and Qvalue < 0.05. TIMER2.0 software was conducted to analyze the stromal and immune cell abundance of the microenvironment indicated by two infiltration scores, stroma score and immune score, in NAFLD-HCC tumor tissue (NAFLD-T), NAFLD-HCC tumor adjacent tissue (NAFLD-L), HBV-HCC tumor tissue (HBV-T), HBV-HCC tumor adjacent tissue (HBV-L) respectively.

### Text-mining

The E-Utilities tool was performed to automatically download NAFLD-HCC or HBV-HCC relevant literatures in batches from PubMed based on these two sets of keywords: (1) (HBV[tiab] OR hepatitis B virus[tiab]) AND (HCC[tiab] OR liver cancer[tiab] OR hepatocellular carcinoma[tiab] OR malignant neoplasm of liver[tiab] OR liver neoplasm[tiab] OR liver carcinoma[tiab]); (2) (HBV[tiab] OR hepatitis B virus[tiab]) AND (HCC[tiab] OR liver cancer[tiab] OR hepatocellular carcinoma[tiab] OR malignant neoplasm of liver[tiab] OR liver neoplasm[tiab] OR liver carcinoma[tiab]). Subsequently, the disease entities of those papers were identified by the PubTator tool and examined by manual curation [17, 18]. We used an explainable gene ontology fingerprint (XGOF) method published in our previous study [17, 19] to automatically produce the knowledge network based on the number of sentences containing specific gene and/or Gene Ontology term in the biomedical literature in a given field, which quantitatively characterizes the association between genes and ontologies. We established the XGOF of NAFLD-HCC and HBC-HCC respectively. We then utilized the CellMarker database [20] to infer the enriched cell types in the NAFLD-HCC or HBV-HCC microenvironment based on XGOF identified genes using multiple hypothesis tests. At last, the enrichment fold was calculated by R clusterProfiler [21], and the enrichment score was defined as -log_2_ (Bonferroni p-value).

### Laboratory experiment validation

We validated the expression of those reported 25 genes that were over-expressed in NAFLD-HCC by the qPCR experiment using independent samples, including 11 NAFLD-HCC and 12 HBV-HCC samples. Furthermore, we validated the two key featured genes, fatty acid-binding protein 4 (FABP4) and Von Willebrand factor (VWF) by another qPCR using additional samples (HBV-L = 11, HBV-T = 11, NAFLD-L = 13, NAFLD-T = 15).

Immunohistochemistry (IHC) staining of two featured genes, FABP4 and VWF, and endothelial cell marker CD31 was conducted in pairs of HCC tumor and adjacent normal liver tissues. IHC staining was assessed using Image-scope software (Aperio Technologies, Inc.), according to the percentage of positively stained cells and staining intensity.

### Prognostic model construction

Cox proportional hazards regression model was used to assess the relationship between multiple factors and patient’s OS time. First, we calculated the coefficients of the full model as follows:

$$\text{OS} \sim {\text{VWF(T/L) + FABP4(T/L) + Age + TNM + Tumorsize + Gender}}$$

The coefficients of age and tumorsize were not significantly different from zero, and the AUC of the full model was not different with that without those two variants significantly. Next, according to an additional LASSO regression, we excluded those two variants in our prediction model. In our model, male was assigned as 1 and female as 0 for gender.

The risk score was calculated using the following formula:

$${\text{Risk score}}\,\;{\text{ = }}\sum {{\text{G * coef N}}}$$

where coef N is the coefficient value, and G is the ratio of NAFLD-T to paired NAFLD-L based on IHC results, TNM and/or gender. Patients were divided into two groups according to the risk score (HRisk, top 50%; LRisk, bottom 50%). Survival analysis was conducted using Kaplan-Meier method. Receiver operating characteristic (ROC) analysis was used to assess the accuracy of model prediction. Nomogram is a robust tool to quantify individual risk in clinical background by integrating multiple risk factors. It was constructed to predict the OS. The point scale in the nomogram was used to assign points to each variable. The calibration curve was drawn to estimate the consistency between actual and predicted survival, and the performance of the model was evaluated by the consistency index (C-index).

### Statistical analysis

Statistical analyses such as the Student’s t-test and Mann Whitney Wilcoxon Test were performed with Prism 8.0 (GraphPad) or R 3.6.3. The correlation between the VWF/FABP4 and CD31 level was calculated by the spearman method. The log-rank test and Cox proportional hazard regression were used to evaluate related predictors of OS in patients with NAFLD-HCC. All p-values less than 0.05 were considered statistically significant.

## Results

### Collection of NAFLD-HCC and HBV-HCC published literature reports

As of August 2022, a total of 2282 papers were initially screened out. Subsequently, the disease entities of those papers were identified by the PubTator tool, and a total of 2,712 NAFLD-HCC reports and 13,514 HBV-HCC reports were obtained after manual curation.

### Cellular composition of the NAFLD-HCC and HBV-HCC Tumor inferred by text-mining and transcriptome data-mining

**Fig. 1:**
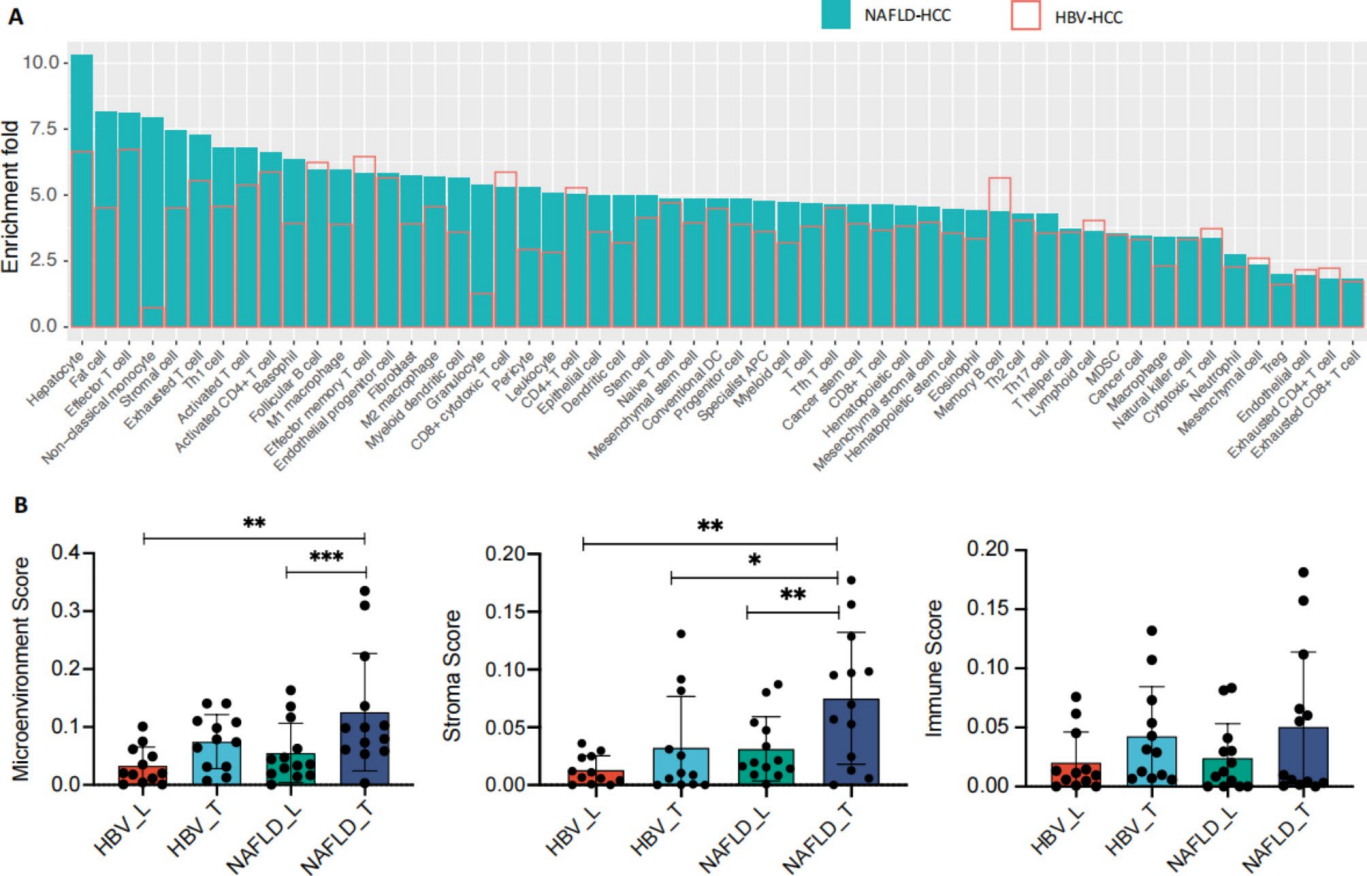
The cellular composition of the NAFLD-HCC and HBV-HCC microenvironment according to text-mining (Fig. 1A**)** and transcriptome data-mining (Fig. 1B) results. (A) The enrichment scores of reported genes in 55 inferred cellular types in the NAFLD-HCC and HBV-HCC respectively according to published literatures using explainable gene ontology fingerprint approach. (B) The microenvironment score, immune score and stroma score of NAFLD-T, NAFLD-L, HBV-T and HBV-L groups calculated by TIMER2.0.

Both text-mining (Fig. 1A**)** and transcriptome data-mining (Fig. 1B) results showed that the enrichment levels of the two HCC microenvironment were different, especially in stromal cells. Through text-mining, we identified 983 NAFLD-HCC related genes, and 1,875 HBV-HCC related genes mentioned in previous publications. Figure 1A showed the enrichment scores of reported genes in 55 inferred cellular types in the NAFLD-HCC and HBV-HCC respectively according to published literatures. The enrichment scores of most cell types in NAFLD-HCC were higher than those in HBV-HCC, such as stromal cells. This result demonstrated that the microenvironmental infiltration of NAFLD-HCC was different from that of HBV-HCC via literature knowledge discovery.

According to transcriptomic RNA sequencing results, the immune cell infiltration analysis revealed that the microenvironment score, immune score and stroma score of NAFLD-T group were generally higher compared with NAFLD-L, HBV-T and HBV-L groups (Fig. 1B). Especially, the stroma score was significantly different between NAFLD-T and the other three groups (all p-values < 0.05).

### Identification of featured genes related to NAFLD-HCC microenvironment

**Fig. 2:**
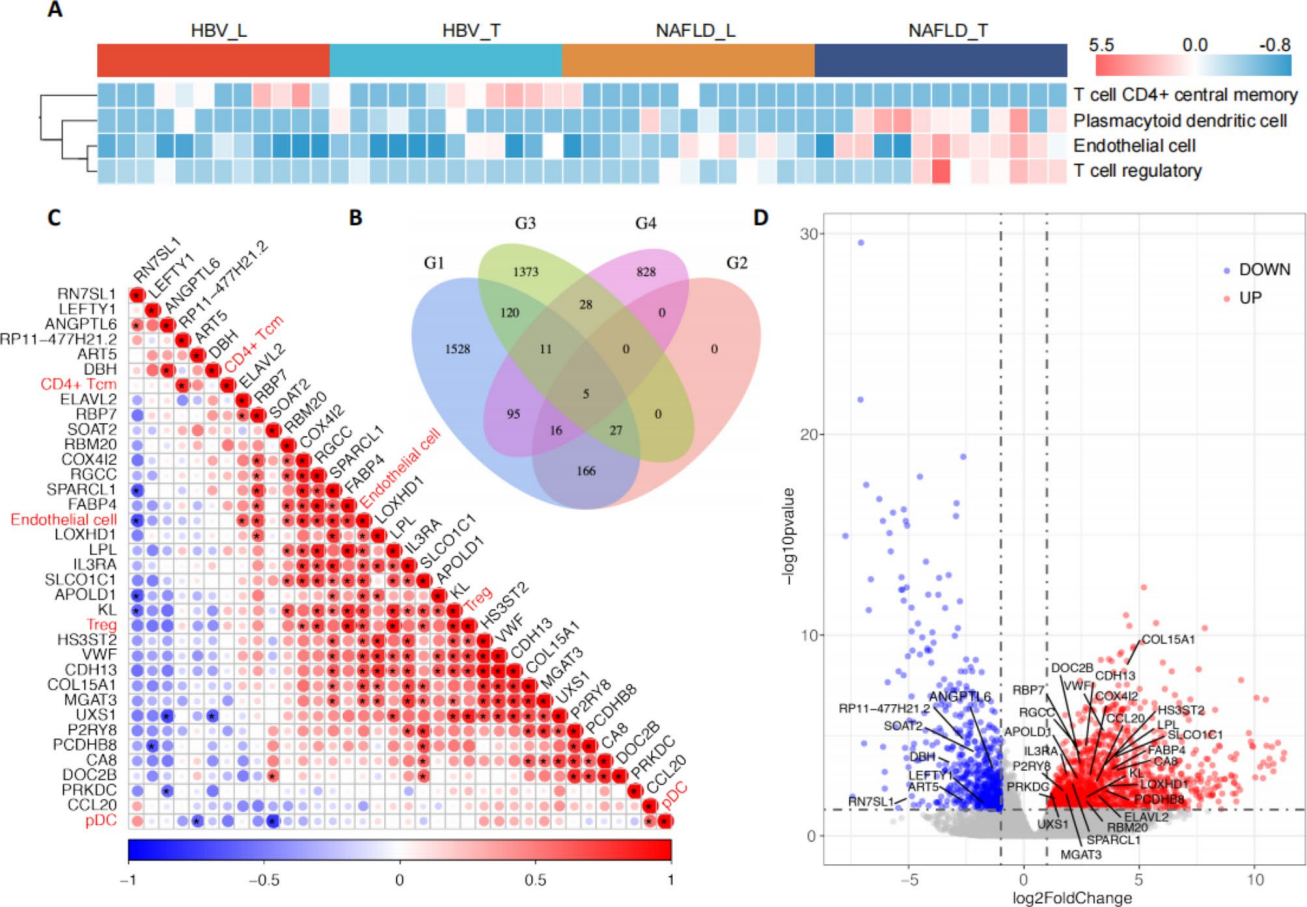
Identification of featured cell types and genes related to NAFLD-HCC microenvironment by transcriptome data A) Four cell types with significantly different infiltration scores including pDC, Treg, CD4^+^ Tcm, and the endothelial cell among NAFLD-T, NAFLD-L, HBV-T and HBV-L groups calculated by TIMER2.0. B) Venn diagram for genes screened by four strategies. G1: 1968 DEGs comparing NAFLD-T and NAFLD-L. G2: 214 genes from G1, whose expression were associated with the cell infiltration scores of four NAFLD-HCC microenvironmental cell types mentioned in Fig. 2A (|r|>0.5, p < 0.05, spearman correlation). G3:1564 DEGs comparing the NAFLD-T and HBV-T. G4: 983 NAFLD-HCC related genes based on previous literatures by text mining C) Correlation among 32 candidate genes (G2 + G3) and the infiltration level of four microenvironmental cells in NAFLD-T. D) Volcano plot for gene expression by RNA-Seq. 32 candidate genes (G2 + G3) associated with the infiltration level of four microenvironmental cells in NAFLD-T were colored. CD4^+^ Tcm: CD4^+^ central memory T cell, Treg: regulatory T cell, pDC: plasmacytoid dendritic cell, DEGs: differential expressed genes

To find the featured genes of the NAFLD-HCC microenvironment, we first screened out the cell types with different abundance according to cell infiltration scores among NAFLD-T samples and the other three types of samples according to TIMER2.0. Figure 2A demonstrated that in the NAFLD-T group, the infiltration scores of the plasmacytoid dendritic cell (pDC), the regulatory T cell (Treg), and the endothelial cell were significantly higher compared with those in NAFLD-L, HBV-T and HBV-L groups, and the score of T cell CD4^+^ central memory cells was much lower. Figure 2B showed different and overlapped genes screened by various strategies including text-mining and transcriptome sequencing methods. The G1 in Fig. 2B represented 1968 DEGs comparing NAFLD-T and NAFLD-L. Among these DEGs in G1, the expression of 214 genes was associated with the cell infiltration scores of four NAFLD-HCC microenvironmental cell types mentioned in Fig. 2A (|r|>0.5, p < 0.05, spearman correlation) (G2). The G3 was 1564 DEGs comparing the NAFLD-T and HBV-T (|log_2_FC|>1, Qvalue < 0.05). The G4 indicated 983 NAFLD-HCC genes based on text mining. Finally, we found a total of 32 candidate genes (G2 + G3) related to the infiltration level of microenvironmental cells in NAFLD-T, of which 5 genes (G2 + G3 + G4) were reported in relevant literatures (Fig. 2C). Among the 5 genes, FABP4 and VWF were overexpressed in the NAFLD-T. The volcano plot depicted that among the 32 genes in NAFLD-HCC, 25 genes were up-regulated and 7 genes were down-regulated (Fig. 2D).

### Experiment validation of the 32 genes differentially expressed in NAFLD-T group

**Fig. 3:**
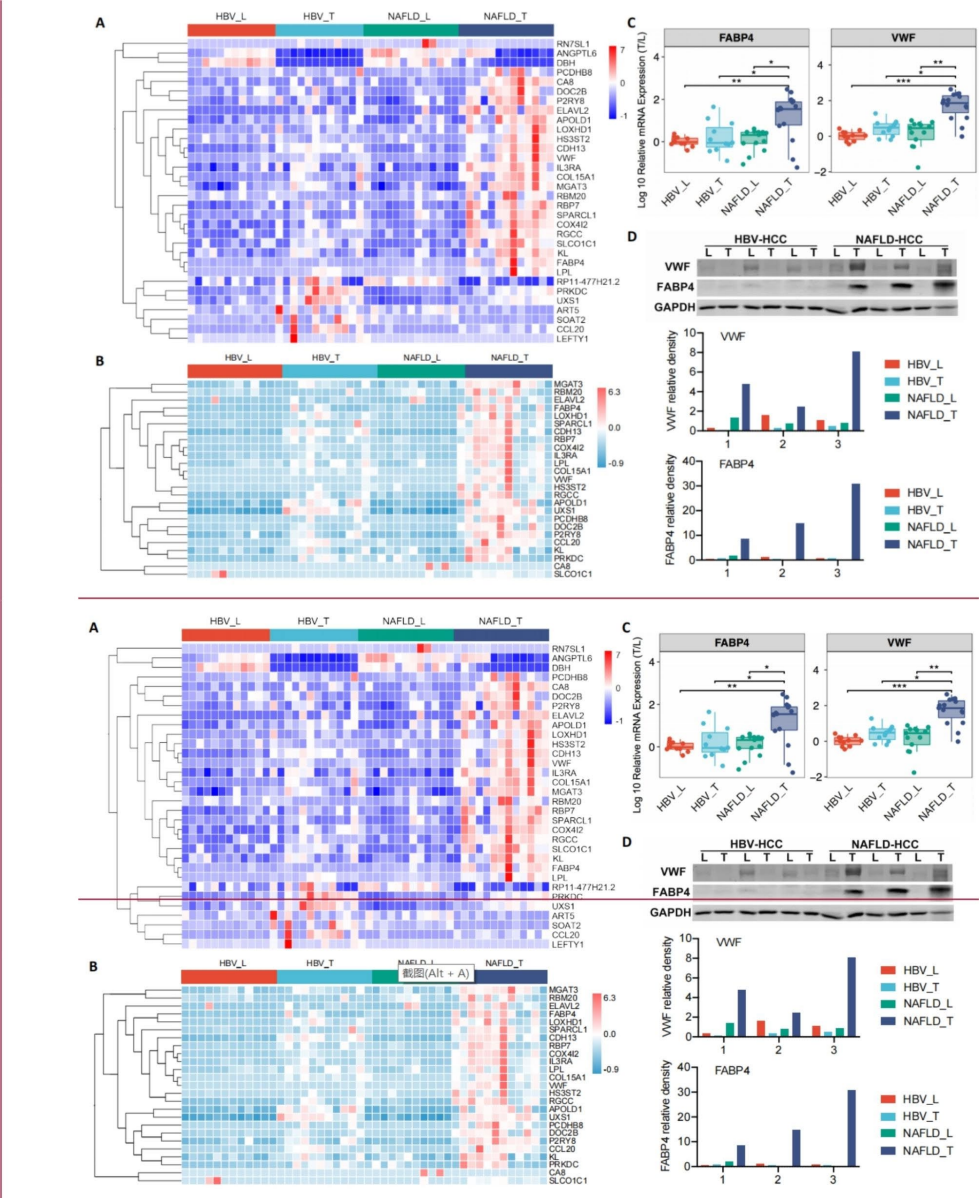
The expression of differential expressed genes (DEGs) between the NAFLD-T and HBV-T which were associated with tumor microenvironment A) Hierarchical clustering of 32 DEGs mentioned in Fig. 2 (G2 + G3). B) Validation of the expression of 25 up-regulated genes by Realtime PCR using 46 samples C) Validation of the expression of FABP4 and VWF by Realtime PCR using 50 samples D) Relative expression of FABP4 and VWF measured by western blot densitometry

**Fig. 4:**
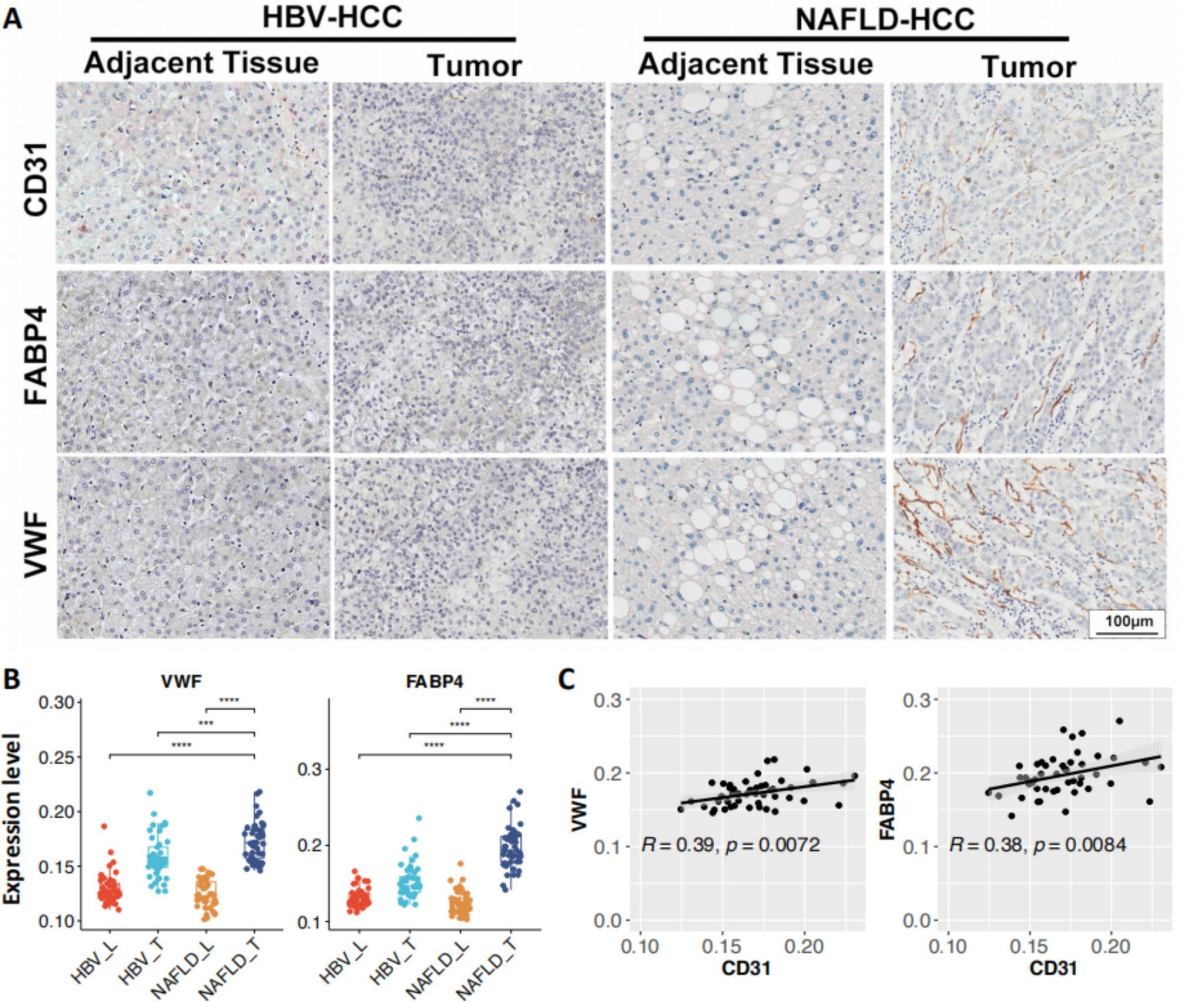
Quantification of VWF, FABP4 and endothelial cell marker CD31 in HBV-HCC and NAFLD-HCC tumor and adjacent normal tissue A) Representative photomicrographs of immunohistochemical staining for VWF, FABP4 and endothelial cell marker CD31 in tumor sections B) Comparison of VWF and FABP4 in HBV-HCC and NAFLD-HCC tumor and adjacent normal tissue according to immunohistochemical staining (***P < 0.005, **** P < 0.001) C) Statistical analysis for the correlation between two NAFLD-HCC featured genes, VWF and FABP4, and CD31 in the immunohistochemical staining

In this study, we validated the expression of those 25 genes that were over-expressed in NAFLD-HCC (Fig. 3A**)** by the qPCR experiment, and we verified that the RNA-level expression of all 25 up-regulated genes were generally consistent with the results of RNA-seq (Fig. 3B). Notably, two text-mining supported genes, FABP4 and VWF, still displayed significantly higher expression in the NAFLD-T group than NAFLD-L in the further validation using another independent sample cohort (HBV-L = 11, HBV-T = 11, NAFLD-L = 13, NAFLD-T = 15, Fig. 3C). The WB experiment also validated that FABP4 and VWF were significantly up-regulated in NAFLD-T at the protein level (all p-values < 0.05, Fig. 3D). The IHC experiment further confirmed that high intensity staining of FABP4 and VWF were both observed in NAFLD-T tissue (Fig. 4A and B), and their levels were positively correlated with CD31 (VWF: r = 0.39, p = 0.0072; FABP4: r = 0.38, p = 0.0084; Fig. 4C). Therefore, these results suggested that FABP4 and VWF were particularly high-expressed within endothelial cells of NAFLD-HCC.

### Prognostic prediction of NAFLD-HCC hepatectomy

The multivariate Cox regression analysis was conducted to determine the effect of the combination of gene sets and clinical indicators on OS. Considering prediction accuracy, multicollinearity, and expression variation among different patients, FABP4 and VWF were included in the prediction model. The results corroborated that combinatorial FABP4, VWF, gender and TNM stage was a significant prognostic factor of NAFLD-HCC (p value = 0.00024) according to the risk score calculation formula:

$$\eqalign{ & - 7.376{\text{ }} \times {\text{}}{\text{VWF(T/L) }} + {\text{ }}5.537{\text{ }} \times {\text{}}{\text{FABP}}4{\text{(T/L) }} & + {\text{ }}21.39{\text{ }} \times \;{\text{Gender}}\; + {\text{ }}23.73{\text{ }} \times {\text{ TNM}} \cr}$$

**Fig. 5:**
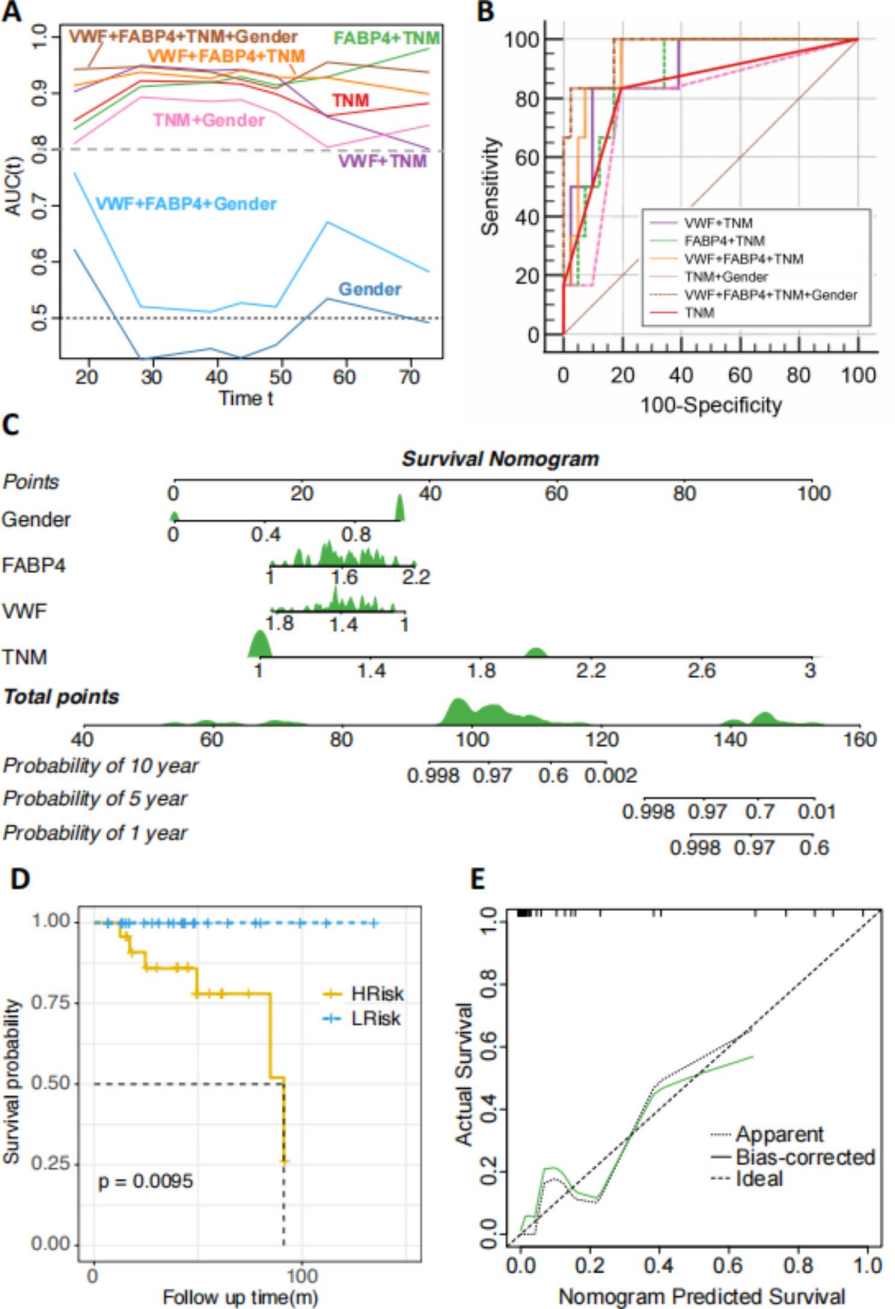
The construction and evaluation of prognostic prediction models for NAFLD-HCC hepatectomy A) Comparisons of VWF and FABP4 with other clinical indicators under the time-dependent ROC for the incidence of overall survival B) ROC of different prognostic prediction models for NAFLD-HCC hepatectomy C) Nomogram for prognostic prediction of NAFLD-HCC hepatectomy. For gender, 1 means male. The distribution of patients was shown in green D) The survival probability of high risk group (HRisk group) and low risk group (LRisk group) E) High consistency indicated by calibration curves between actual and predicted outcomes

Under the time-dependent ROC for the incidence of overall survival, the two-gene sets combined with TNM and gender classifier showed higher accuracy (AUC > 0.9, Fig. 5A). The ROC analysis was used to further evaluate the accuracy of this prognostic model. The value of AUC achieved 0.967 which indicated a good classifier and discriminating ability of the model (Fig. 5B). Moreover, FABP4, VWF, gender and TNM were incorporated to construct a nomogram to predict 1-, 5- and 10-year OS. The consistency index of nomogram was 0.952, which indicated that the nomogram could predict OS with high accuracy (Fig. 5C). The 47 NAFLD-HCC samples were divided into High Risk (HRisk) (top 23 samples) and Low Risk (LRisk) (bottom 24 samples) subgroups sorted by the risk score in descending order. The correlation between two genes was low and their variance inflation factors were less than 10. The model including VWF, FABP4, gender and TNM performed better than that containing only TNM and those consisting of the other combinations. Kaplan–Meier cumulative curve showed a significant difference in OS between the HRisk and LRisk groups. Patients with high-risk scores had worse OS than those with low-risk scores (p-value = 0.0095) (Fig. 5D). In addition, calibration curves indicated high consistency between actual and predicted outcomes (Fig. 5E).

## Discussion

We compared the microenvironments of tumor and adjacent normal samples from HCC patients with either NAFLD or HBV who had tumor resection and different overall survival based on the transcriptome data and follow-up information. We also used text-mining software and biomedical literature data to screen two potential key genes FABP4 and VWF from DEGs. Finally, we built and validated a multi-feature joint model to predict the prognosis of NAFLD-HCC after hepatectomy.

FABP4 is a lipid chaperone protein that binds with high affinity to hydrophobic ligands including saturated and unsaturated long-chain fatty acids. It is responsible for promoting lipid storage, distribution, transportation, decomposition and metabolism [22]. FABP4 is mainly secreted by adipocytes and macrophages. It was elucidated that FABP4, which is not normally expressed in the liver, could be synthesized and secreted by hepatocytes, peritumoral endothelial cells, intra-tumoral hepatic stellate cells, and HCC cells [23]. The up-regulated level of FABP4 in the systemic circulation of patients with NAFLD is associated with liver inflammation and fibrosis [24]. FABP4 could provide fatty acids to malignant cells to maintain cell proliferation and affect cancer progression [25]. A recent study suggested that targeted inhibition of LPL/FABP4/CPT1 fatty acid metabolic axis can effectively prevent the progression of nonalcoholic steatohepatitis to liver cancer [26]. LPL was also screened out in our results, but due to its correlation with FABP4, we did not include it in our prediction model (Fig. 2C). In addition, FABP4 overexpression in intra-tumoral hepatic stellate cells may contribute to hepatocarcinogenesis in patients with metabolic risk factors by modulation of inflammatory pathways [27]. In our study, we also observed an overexpression of FABP4 in NAFLD-HCC tumor tissue. FABP4 may promote liver cancer through endothelial cells in NAFLD-HCC [28]. In another previous study, it was demonstrated that FABP4 in peritumoral endothelial cells from human HCC samples with metabolic syndrome was overexpressed compared with those with other risk factors for chronic liver diseases [28]. This study confirmed the oncogenic role of FABP4 in liver carcinogenesis, highlighting the key role of tumor microenvironment via cross-talks between endothelial and tumors cells mainly through microvesicles release from endothelial cells. This also agreed with our finding that FABP4 is an unfavorable biomarker in the prognosis prediction model, and it is associated with the endothelial cell infiltration score (Fig. 2). However, the effect of FABP4 as the biomarker for HCC is context-dependent. In our study, the level of FABP4 was not different between HBV-HCC tumor and adjacent tissues (P > 0.05). In a previous study from our hospital where most HCC patients carried HBV, FABP4 was low-expressed in tumor tissues compared with the adjacent tissue, and its expression as a favorable biomarker was significantly associated with the tumor size, portal vein tumor thrombus, recurrence-free survival and overall survival [29].

VWF, a multimeric glycoprotein synthesized primarily by endothelial cells, is well known to be involved in angiogenesis and hemostatic mechanisms [30, 31]. The binding of the VWF to integrin avβ3 could repress the VEGFR-2 activity and the downstream pro-angiogenic signaling pathways. The development of HCC is dependent on the formation of new blood vessels, and the surrounding blood vessels play as one type of important tumor microenvironment in tumor initiation and progression in HCC. Growing research evidence suggested that VWF may function as the bivalent mediator of HCC [30]. For example, higher preoperative VWF appeared to be negatively associated with post-resection liver dysfunction in patients with HCC undergoing partial hepatectomy, whereas, high post-resection plasma VWF concentrations indicated the early HCC recurrence [32]. Another study indicated that VWF levels were higher in patients with severe liver fibrosis stage and/or HCC development than in those without [31]. In our study, we found VWF served as a favorable marker for prognosis of NAFLD-HCC after hepatectomy.

Takaya et al. proposed that one possible explanation for these different results was the various progression and underlying causes of liver diseases [31]. For example, cirrhotic patients frequently have hypercoagulability which is associated with elevated VWF and results in markedly increased risk for thromboembolism. Most of the patients in our study had not developed cirrhosis (Table S1), whereas in the study by Takaya et al., all the patients in the case group had developed cirrhosis [31]. This implies the importance to consider the progression and the underlying causes of liver diseases of the patients when using VWF as a biomarker.

A multi-feature prognostic model for NAFLD-related HCC OS was constructed based on transcriptome analysis, follow-up information and text-mining. The risk score which calculated by combining TNM stage, gender and the expression level of FABP4 and VWF, could be used to predict the OS. Previously, one single VWF gene was proposed to predict the stage of HCC [31] or prognosis of HCC after hepatectomy [32]. According to our results, the combination of two key genes, gender and TNM performed better than the other combinations, indicating the advantage of integrating omics data analysis and text-mining.

However, it should be admitted that our research inevitably has some limitations. Firstly, functional experiments are needed to further explore their potential roles and underlying molecular mechanisms. Secondly, due to strict sampling criteria, only 47 patients with NAFLD-HCC undergoing hepatectomy were included. Multi-center clinical studies with larger sample size are expected to verify the results. Thirdly, our model was based on reported NAFLD-HCC related genes, the other DEGs may also play important roles in NAFLD-HCC development and require further exploration.

## Conclusion

The microenvironment feature of NAFLD-HCC was different from that of HBV-HCC based on the transcriptome data. We also screened out two potential key genes FABP4 and VWF related to tumor microenvironment using transcriptome sequencing, follow-up information and biomedical literature data, which were validated by laboratory experiments. Finally, a multi-feature joint model was built and validated to predict the prognosis of NAFLD-HCC after hepatectomy.

### Electronic supplementary material

Below is the link to the electronic supplementary material.

Supplementary Material 1

## Data Availability

The datasets used and/or analysed during the current study are available from the corresponding author on reasonable request.

## Abbreviations

NAFLD: nonalcoholic fatty liver disease
HCC: hepatocellular carcinoma
HBV: hepatitis B virus
HCV: hepatitis C virus
NASH: nonalcoholic steatohepatitis
XGOF: explainable gene ontology fingerprint
DEG: different expressed gene
OS: Overall survival
NAFLD-T: NAFLD-HCC tumor tissue
NAFLD-L: NAFLD-HCC tumor adjacent tissue
HBV-T: HBV-HCC tumor tissue
HBV-L: HBV-HCC tumor adjacent tissue
IHC: Immunohistochemistry
FABP4: fatty acid-binding protein 4
VWF: Von Willebrand factor
ROC: Receiver operating characteristic
C-index: consistency index
HRisk: High Risk
LRisk: Low Risk
pDC: plasmacytoid dendritic cell
Treg: regulatory T cell
AUC: area under the curve
