# Supplementary material for: Novel biomarker genes for the prediction of post-hepatectomy survival of patients with NAFLD-related hepatocellular carcinoma
Source: Cancer Cell Int. 2023 Nov 10;23:269. doi: 10.1186/s12935-023-03106-2 (PMC10638756; doi:10.1186/s12935-023-03106-2)
Supplement: Supplementary file 1 — Supplementary Material 1 [file 12935_2023_3106_MOESM1_ESM.docx]

**Table S1. Baseline characteristics of participants enrolled in the RNA-Seq.**

|  | HBV-HCC | NAFLD-HCC |
| --- | --- | --- |
| Male/Femail | 10/2 | 10/3 |
| Age (SD) year | 47.42 (7.40) | 61.85 (12.88) |
| BMI (SD) kg/m^2^ | 23.79 (1.75) | 29.66 (3.75) |
| Cirrhosis | 4 | 0 |
| Metabolic disorder | 0 hypertension  0 type 2 diabetes  0 gout | 7 hypertension  5 type 2 diabetes  1 gout |
| Maximum tumor size (SD) cm | 8.36 (3.43) | 6.22 (2.08) |
| CA199 (SD) ku/L | 19.18 (12.68) | 27.37 (50.57) |
| AFP (SD) ng/mL | 809.46 (564.69) | 212.03 (429.32) |
| TG (SD) mmol/L | 1.21 (0.97) | 2.52 (1.32) |
| TC (SD) mmol/L | 4.30 (0.61) | 4.83 (0.79) |
| HDL (SD) mmol/L | 1.09 (0.23) | 1.01 (0.22) |
| LDL (SD) mmol/L | 2.95 (0.78) | 3.08 (0.68) |
